# High-Pressure Processing of Kale: Effects on the Extractability, In Vitro Bioaccessibility of Carotenoids & Vitamin E and the Lipophilic Antioxidant Capacity

**DOI:** 10.3390/antiox10111688

**Published:** 2021-10-26

**Authors:** Mario Schmidt, Sofia Hopfhauer, Uwe Schwarzenbolz, Volker Böhm

**Affiliations:** 1 Institute of Nutritional Sciences, Friedrich Schiller University Jena, 07743 Jena, Germany; Mario.Schmidt@uni-jena.de (M.S.); Sofia.Hopfhauer@uni-jena.de (S.H.); 2 Chair of Food Chemistry, Technische Universität Dresden, 01069 Dresden, Germany; Uwe.Schwarzenbolz@tu-dresden.de

**Keywords:** high-pressure processing (HPP), in vitro digestion, digest filtration, α-tocopherol equivalent antioxidant capacity (αTEAC), oxygen radical antioxidant capacity (ORAC)

## Abstract

High pressure processing (HPP) represents a non-thermal preservation technique for the gentle treatment of food products. Information about the impact of HPP on lipophilic food ingredients (e.g., carotenoids, vitamin E) is still limited in more complex matrices such as kale. Both the variation of pressure levels (200–600 MPa) and different holding times (5–40 min) served as HPP parameters. Whereas a slightly decreasing solvent extractability mostly correlated with increasing pressure regimes; the extension of holding times resulted in elevated extract concentrations, particularly at high-pressures up to 600 MPa. Surprisingly, slightly increasing bioaccessibility correlated with both elevated pressures and extended holding times, indicating matrix-dependent processes during in vitro digestion, compared to results of extractability. Moreover, the verification of syringe filters for digest filtration resulted in the highest relative recoveries using cellulose acetate and polyvinylidene difluoride membranes. The α-tocopherol equivalent antioxidant capacity (αTEAC) and oxygen radical antioxidant capacity (ORAC) assays of treated kale samples, chopped larger in size, showed increased antioxidant capacities, regarding elevated pressures and extended holding times. Consequently, one may conclude that HPP was confirmed as a gentle treatment technique for lipophilic micronutrients in kale. Nevertheless, it was indicated that sample pre-treatments could affect HP-related processes in food matrices prior to and possibly after HPP.

## 1. Introduction

Global consumer awareness, in relation to sustainable and clean-living lifestyles, has increased during the last decade. These trends continue to influence demands in the food industry, such as clean labeling and unprocessed food, to preserve health-promoting, bioactive food ingredients [1]. Bioactive compounds can be defined as secondary plant metabolites, which may impact the physiological or cellular activities contributing to health benefits [2]. Vegetables, such as kale (Brassica species), contain natural antioxidants, including hydrophilic vitamin C or polyphenols, as well as lipophilic carotenoids and vitamin E [3]. Moreover, kale was recently reviewed, due to its popularity of being a “superfood” [4]. Reasons for increasing kale consumption may be explained by consumer’s association of kale as part of the cruciferous family with general health aspects, such as the protection against different types of cancers, depending on bioactive ingredients [5,6]. Consequently, the preservation of health-promoting secondary plant metabolites in food is a key to an increased intake via a proper diet.

In contrast to conventional preservation techniques (e.g., pasteurization, sterilization), high-pressure processing (HPP) represents an alternative, non-thermal method to maintain sensory, nutritional, and functional characteristics in minimally processed food, while meeting food safety levels and extended shelf lives [7,8,9]. While previous studies on pressure stabilities of hydrophilic food ingredients (e.g., vitamin C, B group vitamins) were reported for different matrices [10,11,12,13], data for lipophilic micronutrients such as carotenoids and vitamin E in kale are still limited. In general, investigating HPP samples including only one raw material is supposed to improve the understanding of HP processes, prior to an examination of multi-component systems.

Techniques like the solvent extraction of lipophilic compounds, the determination of bioaccessibilities or antioxidant capacities may be utilized to investigate the impact of HP treatments on kale ingredients. Bioaccessibility is defined as the fraction of a compound that is released from its matrix during digestive processes and becomes available for intestinal absorption [14,15]. A previously reported standardization of in vitro digestion assays acts as guideline for further investigations [16]. Where solvent extraction and bioaccessibilities often focus on selected groups of compounds, the determination of antioxidant capacities describes the impact of HPP on all available extracted antioxidants (e.g., phenolic acids, flavonoids, vitamins, carotenoids) as sum parameters, depending on chosen extraction solvents [17,18]. The hydrophilic trolox equivalent antioxidant capacity (TEAC) assay and oxygen radical antioxidant capacity (ORAC) assay represent popular techniques that are based on electron transfer and hydrogen atom transfer reactions. Since TEAC was reported to potentially result in an underestimation of the antioxidant capacity of complex samples, additional ORAC assays may be useful to support or correct for obtained findings [19]. Lipophilic TEAC and ORAC versions may be used to supplement the still limited information on the impact of HPP on lipophilic micronutrients in kale [20,21,22].

Since kale may be used as an ingredient in a variety of potentially HP-treated products, such as spreads, smoothies, and infant food, the aim of this study was the investigation of HPP effects on lipophilic kale ingredients, excluding the examination of storage, in terms of preservative properties. Consequently, the carotenoids, vitamin E, and chlorophylls in kale puree samples were identified and quantified before and after HP treatments. Different pressure parameters (200 MPa, 400 MPa, and 600 MPa) and holding times (5 min, 10 min, and 40 min) were applied to finally express results, in terms of analyte’s extractability. Another purpose was to adapt an in vitro digestion assay for HP-treated kale samples, including an assay characterization, and to report obtained results as bioaccessibility. Finally, determined lipophilic antioxidant capacities were supposed to create supplementary information and a bigger picture by taking the complexity of kale as a food matrix into consideration.

## 2. Materials and Methods

### 2.1. Chemicals

All chemicals were of analytical grade. Solvents for using HPLC, extraction procedures, and to dissolve reference standards were obtained in HPLC grade quality. All aqueous solutions were prepared by using HPLC grade water (18 MΩ) from a Barnstead MicroPure UV system (Thermo Electron LED GmbH, Niederelbert, Germany). Carotenoid standards (97–99%) were purchased from CaroteNature (Münsingen, Switzerland). Pure tocopherols (>95%) were obtained from Calbiochem (Darmstadt, Germany). Pyrogallol (≥99%), magnesium carbonate basic (≥40% as MgO), 2,6-di-*tert*-butyl-4-methyl-phenol (≥99%), and *(all-rac)*-α-tocopheryl acetate (>96%) were purchased from Sigma-Aldrich (Taufkirchen, Germany). Additionally, α-Amylase (≥5 units/mg solid), pepsin (≥250 units/mg solid), and pancreatin (8 × USP) from porcine pancrease were obtained from Sigma-Aldrich. Porcine bile extract was purchased from Santa Cruz Biotechnology (Heidelberg, Germany). Peanut oil was obtained from a local grocery store. Lucantin^®^ Yellow (ethyl-8′-apo-β-caroten-8′-oat) was obtained from BASF SE (Lampertheim, Germany); 2,2′-Azobisisobutyramidinium chloride (98%) and sodium chloride (99.5%) were purchased from Fisher Scientific (Nidderau, Germany). Hydrochloric acid (32%), sodium bicarbonate (≥99.5%), sodium sulfate (≥99%), and dimethyl sulfoxide (≥99.8%) were obtained from Carl Roth GmbH + Co. KG (Karlsruhe, Germany); 2,2′-azino-di-[3-ethylbenzthiazoline sulfonate (6)] (>98%) and pH buffer solutions (pH 4, pH 7, pH 10) were purchased from Sigma-Aldrich. Fluorescein (Reag. Ph. Eur.) was obtained from Riedel-de Haën (Seelze, Germany). Potassium dihydrogen phosphate (≥99.5%) and manganese dioxide (≥90%) were purchased from Merck KGaA (Darmstadt, Germany). Randomly methylated β-cyclodextrin (RMCD) was obtained from TCI Deutschland GmbH (Eschborn, Germany).

### 2.2. Chlorophyll Isolation

A mixture of 1 g of wheat grass powder and oat grass powder was extracted with MeOH/THF (50:50 = *v*/*v*) containing 0.1% butylated hydroxytoluene (BHT). After solvent removal, using a rotary evaporator, the residue was dissolved in 10 mL of *n*-hexane. The isolation of chlorophyll reference standards was achieved via semi-preparative HPLC (Merck Hitachi 7000 series) in normal-phase mode (ACE 5 Sil, 250 × 7.75 mm) using a variable wavelength detector at 662 nm. Gradient elution at 25 °C was used with an *n*-hexane-*i*-propanol eluent (97/3; *v*/*v*) at 0 min, with a linear increase of *i*-propanol (9%) over 60 min, at a flow rate of 4.0 mL/min and injection volume of up to 200 µL sample. Fractions of chlorophyll a and chlorophyll b were isolated at retention times of 22.7 min and 29.9 min, as well as pheophytin at 16.4 min. Identity and purity of isolated fractions was confirmed by reversed-phase HPLC methodology described in Section 2.5. Concentrations of chlorophyll standards were determined via VIS spectrophotometry using wavelengths of 662 nm (chlorophyll a) and 644 nm (chlorophyll b).

### 2.3. Samples Description

Fresh kale was bought during winter from local distributors (Jena, Germany). Kale leaves were manually separated from stems. A knife mill (Retsch Grindomix GM 200) served to shred leaves to puree-like portions at 8000 rpm for 30 s. Kale samples, intended for high-pressure processing, were transferred into 2 mL polypropylene cryo tubes. High-pressure processing was carried out at pressure levels of 200 MPa, 400 MPa, and 600 MPa, including a set of three holding times (5 min, 10 min, and 40 min) for each pressure regime. After HPP treatment, all samples were immediately deep-frozen at −25 °C.

### 2.4. High-Pressure Processing

A high-pressure pilot plant (Dieckers, Willich, Germany) was used for the pressure treatment of kale puree. A mixture of water/ethylene glycol (50:50, *v*/*v*) served as pressure transduction liquid. The unit was equipped with two temperature-controlled 25 mL pressure vessels (Sitec, Maur, Switzerland), which are designed for pressures up to a limit of 700 MPa. Prior to the pressure application, the samples were filled into polypropylene test tubes (2 mL) and wrapped with sealing film. The pressure was increased at the rate of 200 MPa/min, while it was released immediately at the end of the holding time by valve opening. The decompression time was less than 10 s. Kale puree samples were treated for time periods between 5 and 40 min, at pressures up to 600 MPa at RT. All treatments were performed in duplicate and analysed thrice.

### 2.5. Determination of Carotenoids, Vitamin E, and Chlorophyll

#### 2.5.1. Extraction Procedure

Kale samples (0.5 g) were weighed into conical test tubes (50 mL). Then, 200 mg of magnesium carbonate, 200 mg of sodium sulfate, and 25 µL of an internal standard (Lucantin-Yellow^®^, α-tocopheryl acetate) were added. Afterwards, 20 mL of a mixture of MeOH/MtBE (50:50 = *v*/*v*), including 0.1 wt% BHT, served as extraction solvent, following 5 s of vortexing. All samples were then sonicated four times in an ice bath under reduced daylight conditions. Centrifugation at 7000 rpm was applied for phase separation between repetitions of extractions. Consequently, combined upper phases were evaporated under reduced pressure using a rotary evaporator at 30 °C. Afterwards, the residue was dissolved in MeOH/MtBE (70:30 = *v*/*v*), following centrifugation (14,000 rpm, 5 min) for further HPLC analysis.

#### 2.5.2. Identification and Quantification of Carotenoids and Chlorophyll

##### HPLC-DAD

Kale extracts were analyzed using a VWR Hitachi Chromaster (5000 series) reversed-phase HPLC system (Develosil C30, 250 × 4.6 mm, 5 µm, Phenomenex, Aschaffenburg, Germany) at a column temperature of 13 °C and 20 µL injection volume. Both an eluent gradient and a flow gradient were applied. At 0 min, the eluent gradient started at 9% of solvent A (MeOH) and 91% of solvent B (MtBE), at a flow rate of 0.43 mL min^−1^. Solvent A was then increased to 50% over 23.5 min at constant flow rates. Afterwards, solvent A was increased to 70% until 38 min, with an increasing flow rate of 0.6 mL min^−1^, which was held until 40 min. Subsequently, solvent A was reduced to 9%, with an increased flow rate of 1.0 mL min^−1^ and following a holding time of 12 min for equilibration. A diode array detector served for identification, in regards to the characteristic spectral absorbance profiles and quantification of carotenoids (λ_450 nm_), chlorophyll a (λ_662 nm_), and chlorophyll b (λ_644 nm_), in comparison to external standards applying 5-point calibration curves (r > 0.999). Recovery of the internal standard (Lucantin-Yellow^®^) was considered. Chromaster system manager (Version 2.0, Hitachi High-Tech Science Corporation, Tokyo, Japan) was applied for data evaluation.

##### HPLC–MS/MS

Mass spectral analysis was applied to support results from identification via diode array detection. Therefore, a Shimadzu HPLC system (LC-20 series) was hyphenated with a triple quadrupole mass spectrometer (API 2000, AB Sciex). Kale extracts (50 µL) were injected onto a reversed-phase column (YMC C30, 250 × 4.6 mm, 5 µm, YMC Europe, Dinslaken, Germany), applying a gradient elution with MeOH/water (80:20 = *v*/*v*; A) and MtBE/MeOH/water (78:20:2 = *v*/*v*/*v*; B) at 30 °C. Pumping flow mode was kept isocratic at 1.3 mL min^−1^. The gradient elution started with an increase of solvent B to 30% for 5 min, which was increased to 60% until 35 min. Finally, solvent B was set to 100% until 42 min, which was kept for 1 min afterwards. Re-equilibration time was set to 7 min at 100% of solvent A. MS measurements were run in positive Q1 scanning mode, comparing external standards of carotenoids and chlorophylls with compounds from kale extract. Analyst^®^ (Version 1.5.2, AB Sciex, Darmstadt, Germany) was applied for data evaluation.

#### 2.5.3. Identification and Quantification of Vitamin E

Analysis of vitamin E in kale extracts was accomplished via normal-phase chromatography using a Jasco LC-900 series HPLC system and fluorescence detection. Therefore, previously redissolved extract residues in MeOH/MtBE (70:30 = *v*/*v*) were subject to a solvent exchange under nitrogen at 30 °C towards a mixture of *n*-hexane/MtBE (98:2 = *v*/*m*), which was used for isocratic elution, as well. Kale extracts (20 µL) were injected onto an Eurospher Diol column (250 × 4.6 mm, 5 µm, Knauer, Berlin, Germany) with a set flow rate of 1.5 mL min^−1^ at 25 °C for 40 min. The α-tocopherol was identified via comparison of retention times with the corresponding external standard. Quantification was achieved with a 5-point calibration curve (r > 0.999) and taking recovery rates of the internal standard (α-tocopheryl acetate) into account. Excitation and emission wavelengths were set to 292 nm and 330 nm. Jasco ChromNav (Version 1.18.07, Build 3) was applied for data evaluation.

#### 2.5.4. Limits of Detection and Quantification

The limit of detection (LOD) and limit of quantification (LOQ) for each analyte were based on signal-to-noise ratios of S/N = 3:1, as well as S/N = 10:1.

### 2.6. Antioxidant Capacity Assays

#### 2.6.1. Extraction Procedure

A standardized extraction method was established for samples intended for the lipophilic ORAC and TEAC assays. Kale samples (0.5 g) were weighed into 50 mL conical and sealable test tubes. For pre-treatment 2 mL of MeOH were added with a following sonication for 15 min, which was cooled with ice water. Afterwards, 20 mL of *n*-hexane were added with subsequent extraction using a vortex blender for 30 s. Then, test tubes were subjected to centrifugation with 3800 rpm at 4 °C for 1 min. Supernatants were combined in a 250 mL brown round-bottom flask and the extraction procedure was repeated up to 10-fold, until supernatants were colourless. Subsequently, a rotary evaporator was used to remove *n*-hexane under reduced pressure. Residues were dissolved in 5 mL of *n*-hexane afterwards.

#### 2.6.2. The α-Tocopherol Equivalent Antioxidant Capacity (αTEAC) Assay

All experiments were performed according to Müller et al. [20]. Briefly, Trolox, as a reference standard, was replaced by α-tocopherol dissolved in ethanol, for the generation of calibration curves using a U*V*/*V*IS spectrophotometer (V-530, Jasco Deutschland GmbH, Pfungstadt, Germany). An aliquot of 1.5 mL of kale extract in *n*-hexane was transferred into a 2 mL centrifuge tube, with subsequent centrifugation at 14,000 rpm for 5 min at ambient temperature. Afterwards, 100 µL of supernatant were pipetted into a 1.5 mL test tube, followed by the addition of 1000 µL of an aqueous ABTS^•+^ solution (1.6 mM KH_2_PO_4_ buffer at pH 7.4). The test tube was then subject to 30 s of vortexing with subsequent centrifugation for 30 s at 14,000 rpm and ambient temperature. Afterwards, the lower phase was immediately transferred into a disposable 1.6 mL semi-micro cuvette made of polystyrene (Th. Geyer, Renningen, Germany). The subsequent determination of an absorption at 734 nm was initiated after 2 min in total, in relation to first the mixing of the sample and ABTS^•+^ solution. An increasing antioxidant activity correlated with a decreasing absorption value. Blank values consisted of 100 µL of *n*-hexane replacing 100 µL of sample solution.

#### 2.6.3. Lipophilic Oxygen Radical Absorbance Capacity (L-ORAC) Assay

All experiments were performed according to Huang et al. [21] and Watanabe et al. [22]. An aliquot of 1.5 mL of the kale extract in n-hexane was transferred into a 2 mL centrifuge tube with subsequent centrifugation at 14,000 rpm for 5 min at ambient temperature. Afterwards, 750 µL of the supernatant were transferred into a 1.5 mL centrifuge tube, followed by evaporation under a gentle stream of nitrogen at 30 °C. Then, the residue was dissolved in 750 µL of DMSO. Furthermore, a previously prepared aqueous stock solution of β-RMCD (14 wt%) was diluted with acetone (50:50 = *v*/*v*). An aliquot of 750 µL of the obtained β-RMCD solution in acetone was used to dissolve the evaporated extract using a vortex blender. Afterwards, a thermomixer (compact 5350, Eppendorf, Hamburg, Germany) was applied for incubation at 25 °C and 1000 rpm for 1 h. Finally, the incubated mixture was further diluted (50:50 = *v*/*v*) with acetone-containing β-RMCD solution, which was previously spiked with 10 vol% of DMSO. All following steps were performed on a 96-well microplate made from quartz glass. Diluted extracts (50 µL) were combined with 25 µL of fluorescein solution (1.2 µM in 1.6 mM KH_2_PO_4_ buffer at pH 7.4) and 100 µL of phosphate buffer (1.6 mM KH_2_PO_4_ buffer at pH 7.4). Afterwards, the microplate was moved into a filter-based microplate reader (FLUOstar Optima, BMG Labtech GmbH, Ortenberg, Germany) at 37 °C. After 10 min for incubation, the microplate was removed and 150 µL AAPH (129 mM in phosphate buffer at pH 7.4) were immediately added to wells. The following fluorescence measurements were performed at 490 nm (λ_ex_) and 520 nm (λ_em_) at 37 °C and 240 cycles applying a cycle time of 60 sec. Blank values consisted of 50 µL diluted β-RCMD solution in acetone and 10 vol% of DMSO instead of the addition of kale extract. Negative controls were prepared by mixing fluorescein solution (25 µL) with β-RCMD solution containing acetone and DMSO (50 µL) and phosphate buffer (250 µL).

### 2.7. In Vitro Digestion Model

#### 2.7.1. Experimental Design

**Figure 1:**
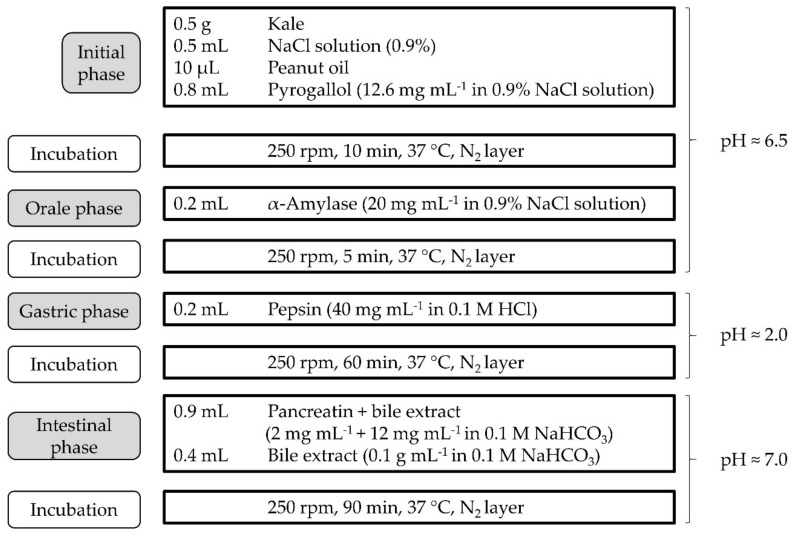
In vitro digestion procedure for determination of bioaccessibility of carotenoids in kale samples.

An in vitro digestion procedure (Figure 1) was adapted from Werner and Böhm [23], Minekus et al. [16], and Reboul et al. [15]. Kale samples were thawed for two hours at ambient temperature in the dark. Conical, sealable test tubes (50 mL, polypropylene) served as vessels for the digestive process. The initial phase was inserted for proper mixing of kale, sodium chloride solution, peanut oil, and pyrogallol serving as antioxidant. An orbital shaker-incubator (Grant-bio ES-20) was operated at 250 rpm and 37 °C under reduced day light conditions. Mixtures of each phase were overlaid with nitrogen for incubation. Adjustment of pH values was achieved by the addition of pre-defined volumes of 0.1 M HCl and 0.1 M NaHCO_3_ solutions containing either enzymes or bile salt, as shown in Figure 1.

#### 2.7.2. Isolation of Micellar Fraction

After completing the digestion process, all samples were subject to centrifugation with 4900 rpm at 4 °C to separate the fraction of released carotenoids from food matrix. Then, each aqueous supernatant was divided into two aliquots, which were transferred to 2 mL test tubes, followed by centrifugation at 14,500 rpm for 5 min at ambient temperature. Supernatants were filtered through a 0.45 µm cellulose acetate filter into sealable 15 mL polypropylene test tubes. Weights of digestion residues and filtered supernatants taken were determined prior to storage at −25 °C for further analysis.

#### 2.7.3. Extraction of Carotenoids and Vitamin E

Applied extraction steps followed a procedure of Werner and Böhm [23], with minor modifications. Previously deep-frozen digestive supernatants and residues were thawed for 2 h at ambient temperature in the dark. First, 2 mL of MeOH, 50 µL of Lucantin-Yellow^®^, 50 µL of α-tocopheryl acetate, and 4 mL of a mixture of MtBE/petroleum ether (50:50 = *v*/*v*) containing 0.1% BHT were added to each supernatant. After 30 s of vortexing, samples were centrifuged for 3 min at 3800 rpm and 10 °C. This extraction process was repeated 4 times in total, and upper phases were combined in brown pear-shaped flasks (100 mL), with adjacent solvent removal under reduced pressure at 30 °C. Remaining residues were dissolved in 5 mL of MeOH/MtBE (70/30 = *v*/*v*) with 0.1% BHT using a sonication bath. Subsequently, aliquots of 1.5 mL were centrifuged at 14,500 rpm for 5 min at ambient temperature for subsequent HPLC-DAD analysis of carotenoids. Finally, an additional aliquot of 400 µL was dried under a gentle stream of nitrogen at 30 °C and dissolved in 400 µL of *n*-hexane/MtBE (98:2 = *v*/*m*), followed by centrifugation at 14,500 rpm for 5 min prior to HPLC analysis of vitamin E. The extraction procedure of digestive residues was modified, in terms of the addition of 1 mL of water prior to the addition of 2 mL of MeOH. Furthermore, the solvent volume MtBE/petroleum ether (50:50 = *v*/*v*) with 0.1% BHT was increased to 8 mL for each extraction cycle.

#### 2.7.4. Calculations

Bioaccessibility was defined as a fraction of carotenoids and vitamin E being potentially available for absorption. Hence, bioaccessibility was calculated in terms of the efficiency of micellization, according to Equation (1):

(1) $$\mathrm{bioaccessibility}\;\left[\%\right]=\frac{\mathrm{content}\;\mathrm{in}\;\mathrm{supernatant}\;\times 100}{\left(\mathrm{content}\;\mathrm{in}\;\mathrm{supernatant}+\mathrm{residue}\right)}$$

The stability of carotenoids and vitamin E during the in-vitro digestion process was determined by comparing contents in the digested samples and raw samples using Equation (2):

(2) $$\mathrm{recovery}\;\left[\%\right]=\frac{\left(\mathrm{content}\;\mathrm{in}\;\mathrm{supernatant}+\mathrm{residue}\right)\times 100}{\mathrm{content}\;\mathrm{in}\;\mathrm{raw}\;\mathrm{sample}}$$

### 2.8. Statistical Analysis

Significance of results was determined by using IBM^®^ SPSS^®^ Statistics (Version 27.0.0.0, Chicago, IL, USA), applying one-factor ANOVA and Tukey-HSD post hoc test (α = 0.05). All results were processed in triplicates.

## 3. Results & Discussion

### 3.1. Identification of Compounds

**Figure 2:**
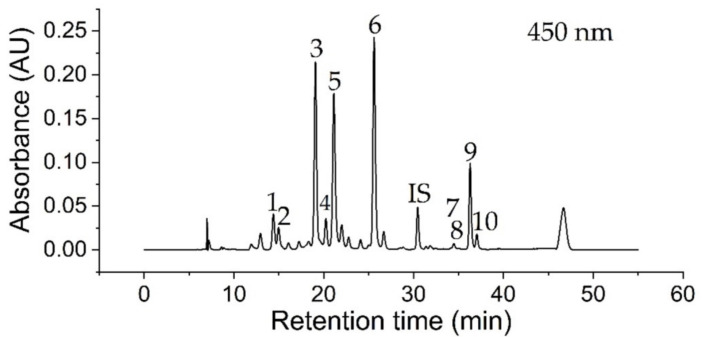
RP-HPLC-DAD chromatogram of a kale extract at 450 nm, using the HPLC parameters described in Section 2.5.2. Identified compounds are listed in Table 1, according to no. 1–10. Lucantin^®^ Yellow was used as internal standard (IS, t_R_ = 30.45 min). A gradient peak caused by LC equilibration appears after 46.7 min.

Carotenoids and chlorophylls were identified in kale extracts according to their retention time, absorption wavelengths, and mass-to-charge ratio via RP-HPLC-DAD/MS measurements presented in Table 1. Acquired data were compared to literature related to carotenoid and chlorophyll content in kale [24]. A representative chromatogram of an extract of untreated (raw, crushed) kale at a wavelength of 450 nm with Lucantin^®^ Yellow as internal standard (IS) is shown in Figure 2. In total, four xanthophylls (peaks 1–4), two chlorophylls (peaks 5, 6) and four carotenes (peaks 7–10) were identified, in relation to characteristic parameters in Table 1. The NP-HPLC-FLD measurements of kale extracts (not shown) resulted in the identification of (all-rac)-α-tocopherol (t_R_ = 8.23 min) using (all-rac)-α-tocopherol acetate as internal standard (t_R_ = 3.04 min). A comparison of identified compounds in kale with literature data confirmed a certain biological diversity between kale species, which can be impacted by environmental influences, such as growing seasons. All identified chemical species except (15Z)-β-carotene were previously reported in literature [25,26,27,28,29,30,31]. Furthermore, γ-tocopherol, α-carotene, α-cryptoxanthin, β-cryptoxanthin, and antheraxanthin were reported as kale ingredients, which could not be identified within this study [27,31].

### 3.2. Extractability

**Figure 3:**
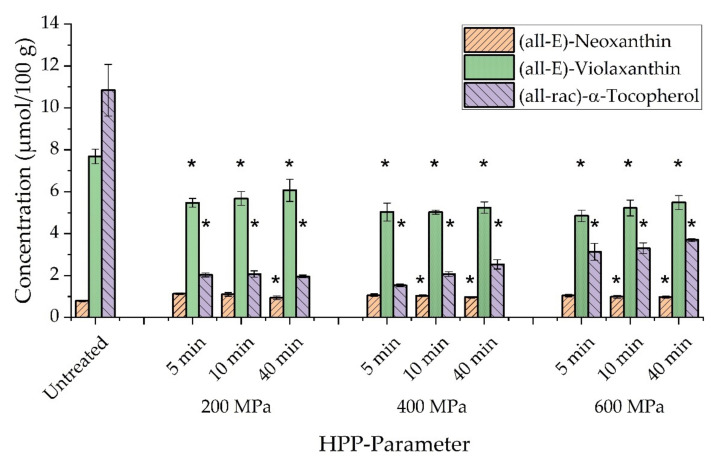
Concentrations of (all-E)-neoxanthin, (all-E)-violaxanthin, and (all-rac)-α-tocopherol in untreated and HP-treated kale in dependence on high-pressure processing (HPP) parameters (200–600 MPa with holding periods of 5 min, 10 min, and 40 min). Untreated kale represents raw, crushed kale samples, without HP-treatment. One-way ANOVA with Tukey-HSD post hoc test; asterisks in the same line indicate significant differences (*p* < 0.05) between treated and untreated samples.

High-pressure processing was previously reported as a gentle preservation technique with no significant or only slight impact on vitamin stability [32]. However, influences on extraction yields were reported, depending on HPP conditions and treated food matrix [33]. Hence, extractability may be used to describe the impact of high-pressure processing on food samples [34,35]. Corresponding results for kale samples are presented in Figure 3. Concentrations of (all-E)-neoxanthin, (all-E)-violaxanthin, and (all-rac)-α-tocopherol in untreated kale were compared to pressurized samples at 200 MPa, 400 MPa, and 600 MPa applying holding periods of 5 min, 10 min, and 40 min.

A decrease in α-tocopherol concentration could be observed for all parameters, compared to untreated kale and depending on HPP conditions. A reduction in concentration of up to 86% was observed at 400 MPa (5 min). However, the effect of sample transport (not shown) between the external HPP site and in-house HPLC analysis accounted for a reduction of up to 60% in α-tocopherol content. Consequently, it might be assumed that sample transport negatively influenced the vitamin E concentration much more than high-pressure processing. Mechanistically, one may suggest a loss of α-tocopherol caused by antioxidant, protective effects towards carotenoids, e.g., β-carotene [36]. A significant decrease in α-tocopherol content (*p* < 0.05) was also reported for HP-treated orange juice-milk samples when pressure conditions exceeded 200 MPa [37]. Non-significant (*p* > 0.05) changes between high pressurized samples and controls were reported for acai juice. However, a significant decrease of α-tocopherol concentration was observed for increasing pressure rates above 500 MPa, by comparison within different pressure regimes of treated acai juice [38]. In contrast, we observed increased vitamin E concentrations in kale correlating with increased pressure and holding periods. Significant differences in α-tocopherol content within pressure parameters were obtained at 600 MPa (10 min, 40 min), in comparison to 200 MPa (5 min). A reverse trend was reported for HP-treated spinach related to a reduced content of α-tocopherol when pressure rates or holding periods were increased [39]. Overall, information about the stability and extractability of vitamin E after HPP is still limited and is often described by either non-significant or only slight changes in concentration [7]. Since α-tocopherol biosynthesis was attributed to the envelope membrane of chloroplasts [40,41,42], it might be assumed that membrane disruption after HPP [43] could partially contribute to an increased concentration of vitamin E in HP-treated kale.

Similar trends could be observed regarding (all-E)-violaxanthin, considering a significant decrease of up to 37%, compared to untreated kale including a decrease of 15% caused by transport. Slightly increasing, but non-significant changes in concentration of (all-E)-violaxanthin were again obtained for elevated pressure rates and extended holding periods, in comparison to an HPP treatment at 200 MPa (5 min). Interestingly, (all-E)-neoxanthin experienced an increase in concentration of 33%, caused by transport of untreated kale, and resulted in decreasing concentrations in correlation to elevated pressure regimes and extended treatment periods, which describes a reversed trend compared to (all-E)-violaxanthin. Significant differences in comparison to untreated kale were found for HPP conditions at 200 MPa (40 min), 400 MPa (10 min, 40 min), and 600 MPa (10 min, 40 min).

Additionally, Table 2 shows concentrations of further carotenes, xanthophylls, and chlorophylls in HP-treated kale samples. No significant differences, compared to untreated kale, could be observed for (all-E)-β-carotene, (9Z)/(13Z)-β-carotene, and (all-E)-lutein. Significantly elevated concentrations were obtained for (15Z)-β-carotene (600 MPa, 40 min), and for all 400 MPa parameters of (all-E)-zeaxanthin including 600 MPa (5 min). Concentrations of chlorophyll a and chlorophyll b decreased significantly after treatments, regarding all pressure parameters and holding periods up to 27% (chl a) and 30% (chl b). However, an increase of carotenoid and chlorophyll concentrations within the pressure regime of 600 MPa could be observed, in relation to the extended holding periods. This trend was partially obtained for pressure treatments at 400 MPa depending on the investigated compound. Interestingly, at 200 MPa, concentrations of (all-E)-β-carotene and (all-E)-lutein did not correlate with an increase of holding periods.

Unaffected concentrations of lutein and β-carotene were reported for HP-treated, freshly prepared broccoli samples, which corresponds to presented results of kale [44]. Nevertheless, previous studies on high-pressure processing of spinach puree showed increased carotenoid and chlorophyll concentrations, which was prepared from previously frozen spinach [39,45]. The present study focussed on HPP treatment of a freshly prepared kale puree though, which did not experience a previous freezing treatment. Elevated chlorophyll concentrations were also reported for HP-treated wheatgrass juice [46]. However, a matrix dependence of carotenoid and chlorophyll concentration in treated vegetables became more obvious in the reported results on HP-treated broccoli, spinach, and green pepper. Therefore, a significantly increased content of lutein was only observed for HP-treated broccoli and spinach, whereas β-carotene concentration increased in spinach only [47]. Apparently, pre-treatments, such as the preparation of juice or puree, may have an impact on carotenoid concentrations of HP-processed carrot juice [48] and carrots [49], wherein β-carotene concentrations were observed as being reduced for juice and unaffected, in the case of carrots. Different trends regarding β-carotene contents were also published for tomato puree [50] and tomato juice [51]. Overall, it might be assumed that a variety of factors could influence the extractability of carotenoids from food matrices. Possible explanations for increased extractabilities after HPP treatment may be the disruption of cellular compartmentalization [52], above 150 MPa, or the occurrence of enzymatic reactions using mild pressure parameters as response to oxidative stress [53,54,55]. Recently, HPP treatment was associated with the accumulation of plant metabolites caused by modulation of gene expression and corresponding coding of biosynthetic enzymes [56]. Both hypothetical mechanisms can be referred to as immediate response (increased extractability) or late response (biosynthetical pathway during storage) after causing stress on plant cells by HPP. However, a change of cellular integrity was also suggested as possible exposure of carotenoids to enzymes, oxygen, and further reactants that cause degradation [48]. Moreover, it was reported that the deposition of carotenoids in vegetables may occur in crystalloid chromoplasts, which are more prone to mechanical stress, compared to globular ones [57]. One may suggest that this could influence the exposure to extracellular room, as well. Furthermore, pressure-resistant plant enzymes, such as lipoxygenase (LOX), may contribute to carotenoid degradation [58,59,60]. Hence, HP-assisted treatments, such as the addition of gentle heating or change in pH value, were suggested to enhance the inactivation of vegetative microorganisms, recently [61].

### 3.3. In Vitro Digestion Assay

#### 3.3.1. Effect of Oil Volume

**Figure 4:**
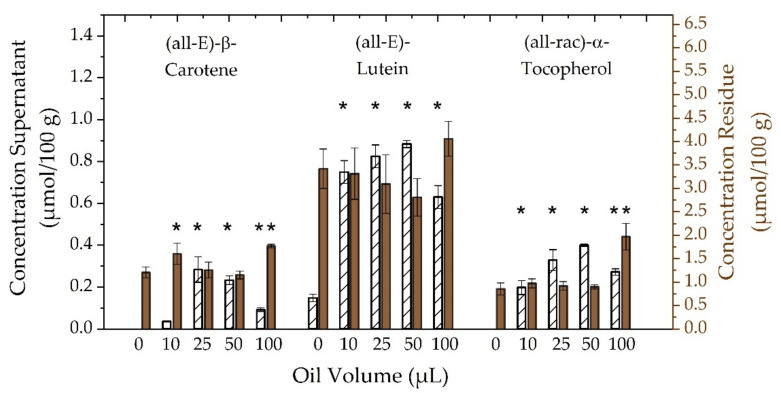
Dependence of concentrations of (all-E)-β-carotene, (all-E)-lutein, and (all-rac)-α-tocopherol after in vitro digestion in aqueous supernatants (left ordinate axis, white bars) and in solid residues (right ordinate axis, brown bars) on added volumes of peanut oil (0–100 µL). One-way ANOVA with Tukey-HSD post hoc test; asterisks in the same line indicate significant differences (*p* < 0.05) between digests with and without oil.

The application of plant oil in digestion assays was reported to potentially impact the bioaccessibility and bioavailability of lipophilic food ingredients [62]. Furthermore, different oil types were investigated, regarding their impact on the availability for intestinal resorption and cell uptake, which was partially referred to the fatty acid composition and ability of pancreatic lipase to bind more easily to mono-saturated fatty acids (MUFAs), compared to poly-saturated fatty acids (PUFAs) [62,63,64]. For this study, peanut oil was chosen as carrier oil, which has both a relatively high amount of MUFAs and low concentrations of α-tocopherol. All in all, little information on added oil volume and the impact on the bioaccessibility of lipophilic micronutrients in digestion assays is known. Consequently, the adapted assay for in vitro digestion of kale was investigated, according to the amount of added peanut oil prior to further applications with highly pressurized kale. In advance, blank digestive experiments without kale have shown that the addition of all selected oil volumes did not affect any carotenoid concentrations of analytes both in supernatants and in residues. Figure 4 shows corresponding results for (all-E)-β-carotene, (all-E)-lutein, and (all-rac)-α-tocopherol of in vitro digestion experiments using kale and different volumes of peanut oil up to 100 µL. All investigated compounds had in common that an increasing amount of oil correlated with an elevated concentration of analytes in aqueous supernatants up to a maximum amount of oil such as 25 µL for (all-E)-β-carotene and 50 µL of oil for (all-E)-lutein and (all-rac)-α-tocopherol. Further increasing the amounts of added oil resulted in decreased analyte concentrations in supernatants. Corresponding reduced concentrations in digest residues confirmed expected trends of reduced analyte contents related to increased oil volumes. This may be attributed to a transfer of compounds from kale samples into the aqueous digestive phase, caused by micellarization. However, the application of highest oil volumes (100 µL) clearly showed technical limitations of the used digestion setup. Analyte concentrations in supernatants decreased, due to oil droplet formation on the vessel walls, which consequently contributed to elevated residue concentration after solvent extraction. Moreover, further loss of analytes could be caused by filtration of oily supernatants. Nevertheless, one needs to consider partially biased results of (all-rac)-α-tocopherol, since peanut oil contains this kind of vitamin E isomer, as well. Hence, increased concentrations in supernatants and residues were also caused by the addition of elevated oil volumes. Overall, the addition of 10 µL of peanut oil showed significantly different (*p* < 0.05) concentrations of all analytes in aqueous supernatants, compared to samples without oil. Especially, (all-E)-β-carotene and (all-rac)-α-tocopherol could not be determined in the aqueous digestive layer without the addition of oil. Finally, the following arguments allowed for choosing 10 µL of peanut oil as default digestion parameter. First, the determined concentration of (all-rac)-α-tocopherol in blank digestion experiments was below the limit of detection. Thus, impacts on results of digested kale samples could be seen as being reduced to a minimum for the applied procedure. Second, according to results for (all-E)-lutein, there were no significant differences (*p* > 0.05) related to larger oil volumes. Moreover, despite a low transfer of (all-E)-β-carotene into the aqueous supernatant, analyte concentrations were above the limit of quantification, which made a further optimization of oil volumes redundant. Additionally, the application of oil volumes larger than 10 µL infrequently required the use of a second syringe filter caused by clogging. This, in turn, may have caused increased standard deviations. Consequently, low oil volumes facilitated a more reliable sample clean up procedure, along with reduced back pressure by syringe filters.

#### 3.3.2. Investigation of Digestion Phases

**Figure 5:**
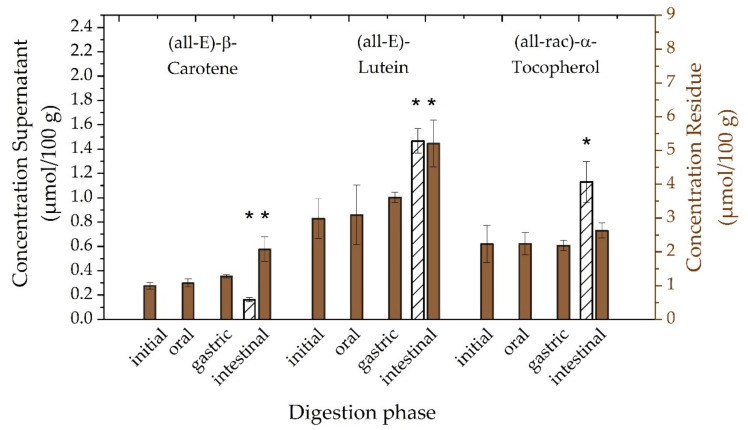
Dependence of concentrations of (all-E)-β-carotene, (all-E)-lutein, and (all-rac)-α-tocopherol after in vitro digestion in aqueous supernatants (left ordinate axis, white bars) and in solid residues (right ordinate axis, brown bars) on digestion phases. One-way ANOVA with Tukey-HSD post hoc test; asterisks in the same line indicate significant differences (*p* < 0.05) between initial phase and oral, gastric, intestinal phases in both supernatant and residue.

Static in vitro digestion models may represent a comparatively simple tool to investigate specific questions in potentially more complex processes. Despite recommendations for performing in vitro digestion, there is a need to choose appropriate conditions according to sample composition. Hence, it might be useful to verify selected digestion parameters and phases related to technical feasibility and reproducibility [16,65]. Consequently, Figure 5 presents the attempt to check the adapted in vitro digestion procedure on potential loss of analytes during initial, oral, gastric, and intestinal phases and, thus, to verify a selected digest parameter. Concentrations of (all-E)-β-carotene, (all-E)-lutein, and (all-rac)-α-tocopherol are represented for supernatants by white bars (left ordinate) and for residues by brown bars (right ordinate). First, no analytes were determined in supernatants until intestinal phase was initiated. This confirms expectations of required additives such as bile salts and pancreatin to allow for micellarization of micronutrients. A relatively low transfer into the aqueous supernatant was observed for (all-E)-β-carotene, compared to (all-E)-lutein. This might be explained by several factors influencing the micellarization of carotenoids, including, but not limited, to carotenoid hydrophobicity. Moreover, it was reported that lutein reduced the transfer of β-carotene into micelle phase [66]. This might be linked to the preferential location of xanthophylls (such as lutein) in the phospholipid surface compared to the triacylglycerol core of lipid droplets, in case of more apolar carotenes [67]. Overall, lower transfer rates were reported for carotenoids in green leafy vegetables caused by association of carotenoids with the light-harvesting complex (thylakoid membrane) in chloroplasts, compared to accumulation in chromoplasts and membrane-bound semicrystalline structures in roots and fruits [68,69,70,71]. Significantly elevated (*p* < 0.05) concentrations of (all-rac)-α-tocopherol in the aqueous supernatant were also determined in comparison to (all-E)-β-carotene. This might be explained by the presence of α-tocopherol mostly either in unbound form in photosynthetic tissues or as fatty acid esters, presumably for the purpose of storage [72,73]. Consequently, one may assume a preferred location of α-tocopherol on the phospholipid surface caused by the hydroxyl group at the chromanol ring and thus showing a behaviour similar to more polar xanthophylls. Furthermore, no significant loss (*p* > 0.05) of analytes in residues was observed comparing all digestive phases with the initial phase. Similar trends were reported for (all-E)-β-carotene and (all-E)-lutein in in vitro digested carrot juice. However, a loss was reported for both compounds in raw spinach after comparison with an untreated sample [74]. Interestingly, significant increases in concentrations of (all-E)-β-carotene and (all-E)-lutein were determined for residues of intestinal phases in comparison to initial, oral, gastric phase (β-carotene), as well as comparing with the initial and oral phase (lutein). An overall increased concentration of (all-E)-lutein might be also explained by its reported relative stability during in vitro digestion linked to the presence of enzymes. In particular, (all-E)-β-carotene was reported to be prone to decay during gastric phases, independent of the presence of enzymes [75].

#### 3.3.3. Filtration of Digest

Sample filtration is an important measure to protect analytical instrumentation, such as HPLC systems. Thereby, several aspects of filter conditions such as hydrophobicity, solvent resistance, adsorption, and further characteristics need to be considered [76]. Hydrophobicity of aqueous supernatants derived from in vitro digestion experiments is difficult to classify, since both hydrophilic and hydrophobic components are present. Information in literature on proper filter selection is limited, and details about chosen filter material are occasionally not given. Since syringe filters of different hydrophobicity were applied for carotenoid analysis in the past, we investigated a variety of materials keeping pore size of 0.45 µm and diameter of 25 mm constant and used one aqueous supernatant for all materials to maintain comparability [14,77,78,79,80]. Concentrations of (all-E)-β-carotene, (all-E)-lutein, and (all-rac)-α-tocopherol after filtration with cellulose acetate (CA), mixed cellulose ester (MCE), polyamide (PA), polypropylene (PP), polytetrafluoroethylene (PTFE), and polyvinylidene difluoride (PVDF) are presented in Table 3. Significant differences (*p* < 0.05) were only observed in case of (all-E)-lutein by comparing PA and PTFE with CA, as well as MCE, PA, and PTFE with PVDF. No significant differences could be determined between CA and PVDF, which at the same time showed highest relative recovery performance, compared to other materials. However, one needs to take into account that recovery, in this case, cannot be considered as absolute value since samples were derived from in vitro digested kale, in contrast to investigating external standards, which can be analyzed without filtration. Furthermore, the addition of echinenone to aqueous supernatants (not shown) confirmed the observed results by showing higher recoveries in case of CA and PVDF, compared to other materials. Finally, relatively more hydrophilic membranes performed better in terms of backpressure during filtration. Hydrophobic materials, such as PP, resulted in challenging sample filtration.

#### 3.3.4. Bioaccessibility

**Figure 6:**
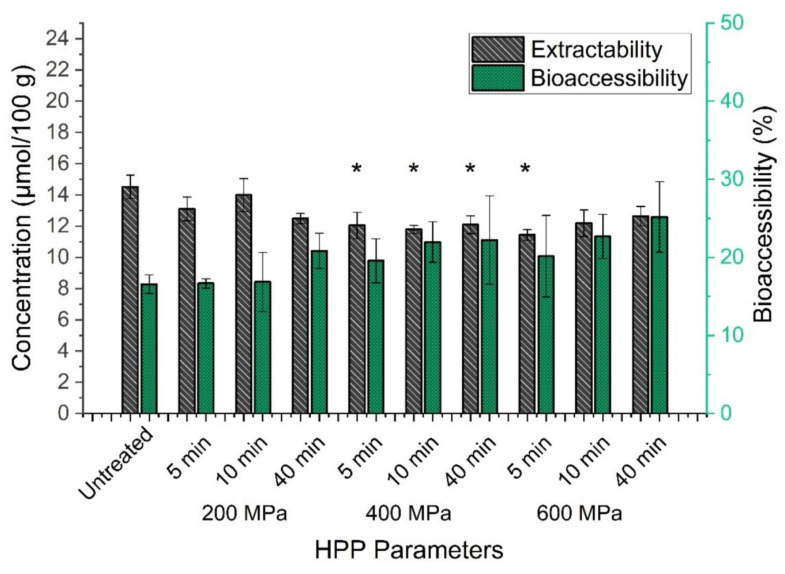
Comparison of extractability (grey bars in µmol/100 g) and bioaccessibility (green bars in %) of (all-E)-lutein in untreated and HP-treated kale using different pressure parameters (200–600 MPa) and holding periods (5–40 min). One-way ANOVA with Tukey-HSD post hoc test; asterisks in the same line indicate significant differences (*p* < 0.05) between treated and untreated samples.

The previous investigation of carotenoid and vitamin E extractability in kale aimed to obtain a quantitative extraction. With these information, conclusions may be drawn related to the effect of high-pressure processing on the total contents of several micronutrients. Additionally, the investigation of bioaccessibilities may lead us to further conclusions on how affected extractabilites may influence the availability of analytes for absorption or later on the assimilation through epithelian tissue and to evaluate potential loss during in vitro digestion [81]. Therefore, in Figure 6 we present a comparison of extractability and bioaccessibility of (all-E)-lutein in kale, depending on applied HPP parameters. Significant differences (*p* < 0.05), compared to untreated kale, were observed for the extractability of HP-treated kale using parameters at 400 MPa (5 min, 10 min, 40 min) and 600 MPa (5 min). Hence, the application of higher pressure rates correlated with decreasing concentrations of (all-E)-lutein whereas the extension of pressure treatments up to 40 min at 600 MPa correlated with slightly elevated concentrations. Although, no significant differences regarding the bioaccessibility of (all-E)-lutein in HP-treated kale samples was observed, an increase of pressure regimes correlated with moderate increasing bioaccessibilities on average. Moreover, extended holding periods during HPP caused elevated bioaccessibilities. This represents a contrary trend, compared to extractability results. Again, this may raise the question about the complexity of competitively occurring reactions both during high-pressure processing and during the in vitro digestion assay. In particular, it may be possible that for example pressure-resistant lipoxygenase (LOX) could lead to carotenoid degradation in digested kale samples treated with lower pressure parameters. It was reported that extremely high-pressure rates of 800 MPa or temperature-assisted HPP at 600 MPa were needed to achieve a complete inactivation of LOX in soybeans. Moreover, elevated treatment periods correlated with reduced LOX activities [82]. Consequently, high residual LOX activities in untreated kale or HP-treated kale at 200 MPa may be associated with increased carotenoids degradation and reduced bioaccessibility in an oil enriched in vitro digestion assay. In contrast, one may ask for possible correlations between reduced LOX activities related to extended HP-treatments at 600 MPa and increased bioaccessibilities of (all-E)-lutein, caused by less LOX-mediated carotenoid degradation during HPP treatment and in vitro digestion. In line with the observed trends of (all-E)-lutein bioaccessibilities, we observed similar results related to (all-E)-β-carotene and (all-rac)-α-tocopherol shown in Table 4. Again, elevated pressures up to 600 MPa and extended holding periods up to 40 min caused slightly increased (but non-significant) bioaccessibilities compared to untreated kale. Highest increases of bioaccessibility compared to untreated kale were observed at 600 MPa for 40 min regarding all investigated compounds. Furthermore, we observed that analyte’s bioaccessibilities were in line with polarity of investigated compounds ((all-E)-lutein > (all-rac)-α-tocopherol > (all-E)-β-carotene). These findings seem to be consistent with other research [83]. On average, recovery values were determined for (all-E)-lutein (24.9 ± 3.3%), (all-E)-β-carotene (34.1 ± 6.9%), and (all-rac)-α-tocopherol (58.5 ± 9.5%).

### 3.4. Antioxidant Capacity

**Figure 7:**
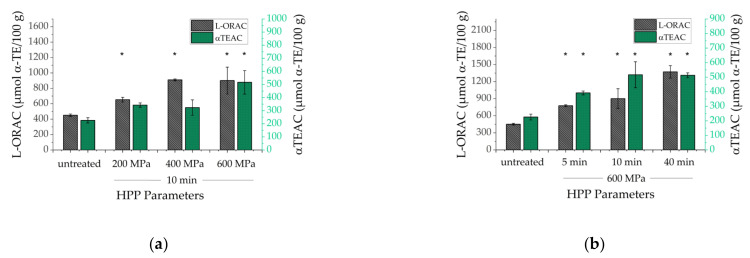
Comparison of the antioxidant capacity of untreated and HP-treated kale, using a fixed treatment period (10 min) and varying pressure rates up to 600 MPa (**a**) as well as a fixed pressure rate (600 MPa) with varying treatment periods up to 40 min (**b**). One-way ANOVA with Tukey-HSD post hoc test: asterisks in the same line indicate significant differences (*p* < 0.05) between treated and untreated samples.

The lipophilic antioxidant capacity (L-AOC) may provide insights about the ability of antioxidants in HP-treated kale samples to scavenge reactive oxygen species and to reduce free radicals [84]. Hence, L-AOC tests such as L-ORAC and αTEAC were applied to investigate the impact of HPP on the entirety of lipophilic antioxidants in treated kale as sum parameter containing all chemical species in contrast to the HPLC analysis of selected carotenoids. Figure 7 shows α-tocopherol equivalents (α-TE µmol/100 g) for both L-ORAC and αTEAC of untreated and HP-treated kale in dependence on different pressure regimes (200 MPa, 400 MPa, 600 MPa) using a fixed duration of 10 min (Figure 7A) as well as holding periods (5 min, 10 min, 40 min) using a pressure regime of 600 MPa, (Figure 7B). It is important to mention that results in Figure 7 need to be considered as independent data set since kale was crushed to larger pieces of approximately 0.5 cm. Hence, leaf structures were partially maintained in contrast to previously presented results using a kale puree in Figure 3. Interestingly, an increase of pressure regimes with a holding period of 10 min correlated with significantly elevated (*p* < 0.05) antioxidant capacities, compared to untreated kale samples. Furthermore, the extension of holding periods using a fixed pressure rate of 600 MPa caused significantly different (*p* < 0.05), increased AOC values compared to untreated kale. Even though one cannot consider AOC results as being only connected to carotenoid extractability, these findings in Figure 7 partially present a contrary trend, compared to carotenoid extractabilities in Figure 3 and Table 2. Extractabilities of carotenoids in treated kale puree mostly decreased after increasing pressure rates. Here, extractabilities increased after increasing pressure regimes (not shown) and resulted in elevated antioxidant capacities, as well. Consequently, one may suggest that kale leaf sizes could have an impact on HP-treated samples, as well. Possibly, the generation of larger pieces of kale leaves caused less cell damage and thus reduced extracellular contact of micronutrients such as carotenoids and chlorophylls to a potentially oxidizing or enzymatically degrading environment prior to HP-treatment. In literature, antioxidant capacities were mostly determined related to hydrophilic plant ingredients in contrast to lipophilic assays used in the present study. Several blended juices such as broccoli and cabbage with apple resulted in increased antioxidant capacities after HPP at 600 MPa [85]. Furthermore, our results are consistent with HP-treated green asparagus (200–600 MPa; 10–20 min), which showed significantly different, elevated AOC values correlating with both higher pressure rates and extended holding periods [86]. Enhanced AOC results were also reported for treated onion samples (100–400 MPa; 5 min) [87]. Furthermore, no impact of HPP on antioxidant capacities were published for treated vegetables such as carrot, tomato, broccoli (500–800 MPa) [88], and tomato juice (200 MPa) [89]. Ultra-high-pressure processing (800 MPa) resulted in both slightly increased and decreased AOC values depending on fruit or vegetables matrices such as orange, orange-lemon-carrot juice, as well as tomato, carrot, and apple [90].

## 4. Conclusions

High-pressure processing does not represent a novel technique for the preservation of food. However, the present study was able to show that HPP may be used as a non-thermal, gentle treatment of minimally processed kale to preserve most lipophilic food ingredients immediately after treatment. Nevertheless, kale represents a complex biological matrix and the results of solvent extractability only reflect a limited class of carotenoids, which were partially affected by slight degradation, as well as increase of concentration. This complicates pin-pointing specific reaction channels to explain sources or sinks of formation and degradation, in order to better understand the processes occurring during HPP treatments. Surprisingly, increasing bioaccessibilities, following increased pressure rates, indicated an opposite trend compared to results of solvent extraction. Consequently, more research may be needed to investigate a possible dependence of the comparability of the extractability and bioaccessibility on biological matrices. Moreover, the impact of food pre-treatments, such as freezing, chopping, and mashing, prior to HPP may be interesting to achieve a better understanding of possible formation and degradation processes of micronutrients in HP-treated food products.

## Figures and Tables

**Table 1:** Identified compounds in kale extracts with experimental parameters derived from external standard measurements.

No.	Compound Name	t_R_ (min)	λ_max 1_ (nm)	λ_max 2_ (nm)	λ_max 3_ (nm)	m/z [M + H]^+^
1	(all-E)-Violaxanthin	14.39	415.0	439.0	469.0	601.3 ^1^
2	(all-E)-Neoxanthin	14.97	413.0	436.0	465.0	601.3 ^2,4^
3	(all-E)-Lutein	19.07	422.0	445.0	473.0	569.2 ^3^
4	(all-E)-Zeaxanthin	20.23	428.0	451.0	478.0	569.2
5	Chlorophyll b	21.12	457.0	589.0	644.0	907.3
6	Chlorophyll a	25.60	432.0	618.0	662.0	893.3
7	(15Z)-β-Carotene	34.13	339.0	449.0	474.0	537.3
8	(13Z)-β-Carotene	34.46	338.0	446.0	470.0	537.3
9	(all-E)-β-Carotene	36.29	-	453.0	479.0	537.3
10	(9Z)-β-Carotene	37.04	-	447.0	473.0	537.3

^1–3^ [M-H_2_O + H]^+^ = 583.2; 583.4; 551.1; ^4^ [M-2H_2_O + H]^+^ = 565.3.

**Table 2:** Concentrations of carotenes, xanthophylls, and chlorophylls in treated and untreated kale in dependence on HPP parameters (200–600 MPa with holding periods of 5 min, 10 min, 40 min). One-way ANOVA with Tukey-HSD post hoc test; asterisks in the same line indicate significant differences (*p* < 0.05) between treated and untreated samples.

Compound	Untreated	200 MPa 5 min	10 min	40 min	400 MPa 5 min	10 min	40 min	600 MPa 5 min	10 min	40 min
Concentration in µmol/100 g										
(all-E)-β-Carotene	9.83 ± 0.53	9.66 ± 0.69	9.36 ± 0.33	10.20 ± 0.90	8.81 ± 0.52	8.53 ± 0.23	8.60 ± 0.37	8.14 ± 0.16	8.73 ± 0.78	9.67 ± 0.83
(9Z)-β-Carotene	1.90 ± 0.15	2.11 ± 0.12	2.06 ± 0.18	1.02 ± 0.01	1.73 ± 0.09	1.76 ± 0.04	1.81 ± 0.08	1.74 ± 0.09	1.85 ± 0.01	2.03 ± 0.12
(13Z)-β-Carotene	0.92 ± 0.06	0.81 ± 0.05	0.83 ± 0.01	0.90 ± 0.12	0.91 ± 0.03	0.94 ± 0.07	0.98 ± 0.06	0.74 ± 0.08	0.88 ± 0.11	1.05 ± 0.05
(15Z)-β-Carotene	0.16 ± 0.01	0.18 ± 0.01	0.21 ± 0.01	0.23 ± 0.04	0.19 ± 0.02	0.21 ± 0.01	0.22 ± 0.03	0.23 ± 0.01	0.23 ± 0.02	0.27 ± 0.03 *
(all-E)-Lutein	14.51 ± 0.75	13.11 ± 0.75	14.00 ± 1.06	12.49 ± 0.33	12.05 ± 0.85	11.79 ± 0.25	12.09 ± 0.57	11.44 ± 0.35	12.19 ± 0.84	12.64 ± 0.63
(all-E)-Zeaxanthin	0.84 ± 0.04	0.94 ± 0.01	0.99 ± 0.07	1.02 ± 0.08	0.93 ± 0.03 *	0.96 ± 0.08 *	0.97 ± 0.12 *	0.87 ± 0.06 *	0.91 ± 0.09	0.95 ± 0.03
Chlorophyll a	77.99 ± 5.28	64.93 ± 2.04 *	65.31 ± 2.00 *	69.62 ± 6.52	57.04 ± 1.47 *	58.87 ± 3.69 *	61.08 ± 3.51 *	56.94 ± 2.53 *	62.14 ± 4.84 *	63.19 ± 2.81 *
Chlorophyll b	27.78 ± 2.42	22.71 ± 0.42 *	24.44 ± 2.62	23.31 ± 0.73	20.84 ± 1.43 *	20.79 ± 0.64 *	22.02 ± 1.48 *	19.23 ± 1.46 *	21.05 ± 1.87 *	21.60 ± 0.23 *

**Table 3:** Concentrations of (all-E)-β-carotene, (all-E)-lutein, and (all-rac)-α-tocopherol after filtration of an aqueous supernatant derived from in vitro digestion of kale using different syringe membrane materials (0.45 µm pore size, 25 mm diameter). Tested membrane materials consisted of cellulose acetate (CA), mixed cellulose ester (MCE), polyamide (PA), polypropylene (PP), polytetrafluoroethylene (PTFE), and polyvinylidene difluoride (PVDF).

Filter Material						
Compound	CA	MCE	PA	PP	PTFE	PVDF
	Concentration in Filtered Supernatant (µmol/100 g)					
(all-E)-β-Carotene	0.19 ± 1.5 × 10^−5^	0.20 ± 2.3 × 10^−3^	0.17 ± 7.0 × 10^−4^	0.18 ± 0.04	0.17 ± 7.8 × 10^−4^	0.20 ± 0.01
(all-E)-Lutein	0.51 ± 0.01	0.49 ± 7.6 × 10^−4^	0.44 ± 3.6 × 10^−3^	0.50 ± 0.01	0.45 ± 0.02	0.52 ± 0.01
α-Tocopherol	0.40 ± 0.01	0.39 ± 0.01	0.36 ± 0.01	0.36 ± 0.04	0.38 ± 0.02	0.39 ± 0.02

**Table 4:** Bioaccessibility of (all-E)-β-carotene and (all-rac)-α-tocopherol in untreated and HP-treated kale in dependence on HPP parameters (200–600 MPa with holding periods of 5 min, 10 min, and 40 min).

Compound	Un- treated	200 MPa 5 min	10 min	40 min	400 MPa 5 min	10 min	40 min	600 MPa 5 min	10 min	40 min
Bioaccessibility in %										
β-Carotene	1.4 ± 0.2	1.5 ± 0.1	1.3 ± 0.3	1.6 ± 0.3	2.0 ± 0.5	2.0 ± 0.3	2.1 ± 0.6	2.1 ± 0.6	2.2 ± 0.3	2.5 ± 0.4
α-Toco- pherol	13.0 ± 1.5	11.3 ± 4.2	14.0 ± 5.6	14.7 ± 2.9	16.7 ± 5.9	17.4 ± 3.8	20.1 ± 6.2	17.9 ± 3.3	19.1 ± 4.3	21.0 ± 2.1

## Data Availability

All data generated or analyzed during this study are included in this article.

## References

[1] Arenas-Jal M., Suñé-Negre J.M., Pérez-Lozano P., García-Montoya E. (2020). Trends in the food and sports nutrition industry: A review. Crit. Rev. Food Sci. Nutr..

[2] Kris-Etherton P.M., Lefevre M., Beecher G.R., Gross M.D., Keen C.L., Etherton T.D. (2004). Bioactive compounds in nutrition and health-research methodologies for establishing biological function: The antioxidant and anti-inflammatory effects of flavonoids on atherosclerosis. Annu. Rev. Nutr..

[3] Podsędek A. (2007). Natural antioxidants and antioxidant capacity of brassica vegetables: A review. LWT Food Sci. Technol..

[4] Šamec D., Urlić B., Salopek-Sondi B. (2019). Kale (brassica oleracea var. acephala) as a superfood: Review of the scientific evidence behind the statement. Crit. Rev. Food Sci. Nutr..

[5] Dias J.S. (2012). Nutritional quality and health benefits of vegetables: A review. Food Nutr. Sci..

[6] Dias J.S. (2012). Major Classes of phytonutriceuticals in vegetables and health benefits: A review. J. Nutr. Ther..

[7] Mahadevan S., Karwe M.V., Balasubramaniam V.M., Barbosa-Cánovas G.V., Lelieveld H.L.M. (2016). Effect of high-pressure processing on bioactive compounds. High Pressure Processing of Food.

[8] Knorr D. (1993). Effects of high-hydrostatic-pressure processes on food safety and quality. Food Technol..

[9] Bello E., Martínez G., Ceberio B., Rodrigo D., López A. (2014). High pressure treatment in foods. Foods.

[10] Tewari S., Sehrawat R., Nema P.K., Kaur B.P. (2017). Preservation effect of high pressure processing on ascorbic acid of fruits and vegetables: A review. J. Food Biochem..

[11] Taoukis P.S., Panagiotidis P., Stoforos N.G., Butz P., Fister H., Tauscher B. (1998). Kinetics of vitamin C degradation under high pressure–moderate temperature processing in model systems and fruit juices. High Pressure Food Science, Bioscience and Chemistry.

[12] Eshtiaghi M.N., Knorr D. (1993). Potato cubes response to water blanching and high hydrostatic pressure. J. Food Sci..

[13] Quaglia G.B., Gravina R., Paperi R., Paoletti F. (1996). Effect of high pressure treatments on peroxidase activity, ascorbic acid content and texture in green peas. LWT Food Sci. Technol..

[14] Hedrén E., Diaz V., Svanberg U. (2002). Estimation of carotenoid accessibility from carrots determined by an in vitro digestion method. Eur. J. Clin. Nutr..

[15] Reboul E., Richelle M., Perrot E., Desmoulins-Malezet C., Pirisi V., Borel P. (2006). Bioaccessibility of carotenoids and vitamin E from their main dietary sources. J. Agric. Food Chem..

[16] Minekus M., Alminger M., Alvito P., Ballance S., Bohn T., Bourlieu C., Carrière F., Boutrou R., Corredig M., Dupont D. (2014). A standardised static in vitro digestion method suitable for food—An international consensus. Food Funct..

[17] Munteanu I.G., Apetrei C. (2021). Analytical methods used in determining antioxidant activity: A review. Int. J. Mol. Sci..

[18] Huang D., Ou B., Prior R.L. (2005). The chemistry behind antioxidant capacity assays. J. Agric. Food Chem..

[19] Zulueta A., Esteve M.J., Frígola A. (2009). ORAC and TEAC assays comparison to measure the antioxidant capacity of food products. Food Chem..

[20] Müller L., Theile K., Böhm V. (2010). In vitro antioxidant activity of tocopherols and tocotrienols and comparison of vitamin E concentration and lipophilic antioxidant capacity in human plasma. Mol. Nutr. Food Res..

[21] Huang D., Ou B., Hampsch-Woodill M., Flanagan J.A., Deemer E.K. (2002). Development and validation of oxygen radical absorbance capacity assay for lipophilic antioxidants using randomly methylated β-cyclodextrin as the solubility enhancer. J. Agric. Food Chem..

[22] Watanabe J., Oki T., Takebayashi J., Yamasaki K., Takano-Ishikawa Y., Hino A., Yasui A. (2013). Improvement of the lipophilic-oxygen radical absorbance capacity (L-ORAC) method and single-laboratory validation. Biosci. Biotechnol. Biochem..

[23] Werner S., Böhm V. (2011). Bioaccessibility of carotenoids and vitamin E from pasta: Evaluation of an in vitro digestion model. J. Agric. Food Chem..

[24] Britton G., Liaaen-Jensen S., Pfander H. (2004). Carotenoids: Handbook.

[25] De Azevedo C.H., Rodriguez-Amaya D.B. (2005). Carotenoid composition of kale as influenced by maturity, season and minimal processing. J. Sci. Food Agric..

[26] Lefsrud M., Kopsell D., Wenzel A., Sheehan J. (2007). Changes in kale (brassica oleracea l. var. acephala) carotenoid and chlorophyll pigment concentrations during leaf ontogeny. Sci. Hort..

[27] Müller H. (1997). Determination of the carotenoid content in selected vegetables and fruit by HPLC and photodiode array detection. Eur. Food Res. Technol. A.

[28] Huck C.W., Popp M., Scherz H., Bonn G.K. (2000). Development and evaluation of a new method for the determination of the carotenoid content in selected vegetables by HPLC and HPLC-MS-MS. J. Chromatogr. Sci..

[29] Park S.-Y., Choi S.R., Lim S.-H., Yeo Y., Kweon S.J., Bae Y.-S., Kim K.W., Im K.-H., Ahn S.K., Ha S.-H. (2014). Identification and quantification of carotenoids in paprika fruits and cabbage, kale, and lettuce leaves. J. Korean Soc. Appl. Biol. Chem..

[30] Becerra-Moreno A., Alanís-Garza P.A., Mora-Nieves J.L., Mora-Mora J.P., Jacobo-Velázquez D.A. (2014). Kale: An excellent source of vitamin C, pro-vitamin A, lutein and glucosinolates. CyTA J. Food.

[31] Kurilich A.C., Tsau G.J., Brown A., Howard L., Klein B.P., Jeffery E.H., Kushad M., Wallig M.A., Juvik J.A. (1999). Carotene, tocopherol, and ascorbate contents in subspecies of *brassica oleracea*. J. Agric. Food Chem..

[32] Oey I., Van der Plancken I., Van Loey A., Hendrickx M. (2008). Does high pressure processing influence nutritional aspects of plant based food systems?. Trends Food Sci. Technol..

[33] Jung S. (2016). Applications and opportunities for pressure-assisted extraction. High Pressure Processing of Food: Principles, Technology and Applications.

[34] De Ancos B., Rodrigo M.J., Sánchez-Moreno C., Pilar Cano M., Zacarías L. (2020). Effect of high-pressure processing applied as pretreatment on carotenoids, flavonoids and vitamin C in juice of the sweet oranges “Navel” and the red-fleshed “Cara Cara”. Food Res. Int..

[35] Hernández-Carrión M., Vázquez-Gutiérrez J.L., Hernando I., Quiles A. (2014). Impact of high hydrostatic pressure and pasteurization on the structure and the extractability of bioactive compounds of persimmon “Rojo Brillante”. J. Food Sci..

[36] Böhm F., Edge R., Land E.J., McGarvey D.J., Truscott T.G. (1997). Carotenoids enhance vitamin E antioxidant efficiency. J. Am. Chem. Soc..

[37] Barba F.J., Esteve M.J., Frigola A. (2012). Impact of high-pressure processing on vitamin E (α-, γ-, and δ-tocopherol), vitamin D (cholecalciferol and ergocalciferol), and fatty acid profiles in liquid foods. J. Agric. Food Chem..

[38] Da Silveira T.F.F., Cristianini M., Kuhnle G.G., Ribeiro A.B., Filho J.T., Godoy H.T. (2019). Anthocyanins, non-anthocyanin phenolics, tocopherols and antioxidant capacity of açaí juice (euterpe oleracea) as affected by high pressure processing and thermal pasteurization. Innov. Food Sci. Emerg. Technol..

[39] Westphal A., Schwarzenbolz U., Böhm V. (2018). Effects of high pressure processing on bioactive compounds in spinach and rosehip puree. Eur. Food Res. Technol..

[40] Schultz G., Bickel H., Buchholz B., Soll J., Akoyunoglou G. (1981). Photosynthesis: Proceedings of the fifth International Congress on Photosynthesis, 7–13 September 1980, Halkidiki, Greece.

[41] Mene-Saffrane L. (2017). Vitamin E biosynthesis and its regulation in plants. Antioxidants.

[42] Mokrosnop V.M. (2014). Functions of tocopherols in the cells of plants and other photosynthetic organisms. Ukr. Biochem. J..

[43] Zhang L., Yao J., Zhang Y., Liao X., Chen F., Hu X. (2015). Microstructural and morphological behaviors of asparagus lettuce cells subject to high pressure processing. Food Res. Int..

[44] McInerney J.K., Seccafien C.A., Stewart C.M., Bird A.R. (2007). Effects of high pressure processing on antioxidant activity, and total carotenoid content and availability, in vegetables. Innov. Food Sci. Emerg. Technol..

[45] Arnold C., Schwarzenbolz U., Böhm V. (2014). Carotenoids and chlorophylls in processed xanthophyll-rich food. LWT Food Sci. Technol..

[46] Ali N., Popović V., Koutchma T., Warriner K., Zhu Y. (2020). Effect of thermal, high hydrostatic pressure, and ultraviolet-C processing on the microbial inactivation, vitamins, chlorophyll, antioxidants, enzyme activity, and color of wheatgrass juice. J. Food Process. Eng..

[47] Sánchez C., Baranda A.B., Martínez de Marañón I. (2014). The effect of high pressure and high temperature processing on carotenoids and chlorophylls content in some vegetables. Food Chem..

[48] Stinco C.M., Szczepańska J., Marszałek K., Pinto C.A., Inácio R.S., Mapelli-Brahm P., Barba F.J., Lorenzo J.M., Saraiva J.A., Meléndez-Martínez A.J. (2019). Effect of high-pressure processing on carotenoids profile, colour, microbial and enzymatic stability of cloudy carrot juice. Food Chem..

[49] Vervoort L., Van der Plancken I., Grauwet T., Verlinde P., Matser A., Hendrickx M., Van Loey A. (2012). Thermal versus high pressure processing of carrots: A comparative pilot-scale study on equivalent basis. Innov. Food Sci. Emerg. Technol..

[50] Jeż M., Błaszczak W., Zielińska D., Wiczkowski W., Białobrzewski I. (2020). Carotenoids and lipophilic antioxidant capacities of tomato purées as affected by high hydrostatic pressure processing. Int. J. Food Sci. Technol..

[51] Yan B., Martínez-Monteagudo S.I., Cooperstone J.L., Riedl K.M., Schwartz S.J., Balasubramaniam V.M. (2017). Impact of thermal and pressure-based technologies on carotenoid retention and quality attributes in tomato juice. Food Bioprocess. Technol..

[52] Gonzalez M.E., Jernstedt J.A., Slaughter D.C., Barrett D.M. (2010). Influence of cell integrity on textural properties of raw, high pressure, and thermally processed onions. J. Food Sci..

[53] Serment-Moreno V., Jacobo-Velázquez D.A., Torres J.A., Welti-Chanes J. (2017). Microstructural and physiological changes in plant cell induced by pressure: Their role on the availability and pressure-temperature stability of phytochemicals. Food Eng. Rev..

[54] Jacobo-Velázquez D.A., del Cuéllar-Villarreal M.R., Welti-Chanes J., Cisneros-Zevallos L., Ramos-Parra P.A., Hernández-Brenes C. (2017). Nonthermal processing technologies as elicitors to induce the biosynthesis and accumulation of nutraceuticals in plant foods. Trends Food Sci. Technol..

[55] Hu K., Peng D., Wang L., Liu H., Xie B., Sun Z. (2021). Effect of mild high hydrostatic pressure treatments on physiological and physicochemical characteristics and carotenoid biosynthesis in postharvest mango. Postharvest Biol. Technol..

[56] Ramos-Parra P.A., García-Salinas C., Rodríguez-López C.E., García N., García-Rivas G., Hernández-Brenes C., Díaz de la Garza R.I. (2019). High hydrostatic pressure treatments trigger de novo carotenoid biosynthesis in papaya fruit (carica papaya cv. maradol). Food Chem..

[57] Panozzo A., Lemmens L., Van Loey A., Manzocco L., Nicoli M.C., Hendrickx M. (2013). Microstructure and bioaccessibility of different carotenoid species as affected by high pressure homogenisation: A case study on differently coloured tomatoes. Food Chem..

[58] Ortega-Rivas E. (2010). Processing Effects on Safety and Quality of Foods.

[59] Chedea V.S., Jisaka M. (2013). Lipoxygenase and carotenoids: A co-oxidation story. Afr. J. Biotechnol..

[60] Biacs P.A., Daood H.G. (2000). Lipoxygenase-catalysed degradation of carotenoids from tomato in the presence of antioxidant vitamins. Biochem. Soc. Trans..

[61] Yang P., Rao L., Zhao L., Wu X., Wang Y., Liao X. (2021). High pressure processing combined with selected hurdles: Enhancement in the inactivation of vegetative microorganisms. Compr. Rev. Food Sci. Food Saf..

[62] Huo T., Ferruzzi M.G., Schwartz S.J., Failla M.L. (2007). Impact of fatty acyl composition and quantity of triglycerides on bioaccessibility of dietary carotenoids. J. Agric. Food Chem..

[63] Xia Z., Han Y., Du H., McClements D.J., Tang Z., Xiao H. (2020). Exploring the effects of carrier oil type on in vitro bioavailability of β-carotene: A cell culture study of carotenoid-enriched nanoemulsions. LWT.

[64] Alberdi-Cedeño J., Ibargoitia M.L., Guillén M.D. (2020). Study of the in vitro digestion of olive oil enriched or not with antioxidant phenolic compounds. Relationships between bioaccessibility of main components of different oils and their composition. Antioxidants.

[65] Alegría A., Garcia-Llatas G., Cilla A., Verhoeckx K., Cotter P., López-Expósito I., Kleiveland C., Lea T., Mackie A., Requena T., Swiatecka D., Wichers H. (2015). Static digestion models: General introduction. The Impact of Food Bioactives on Health: In Vitro and Ex Vivo Models.

[66] Tyssandier V., Lyan B., Borel P. (2001). Main factors governing the transfer of carotenoids from emulsion lipid droplets to micelles. Biochim. Biophys. Acta Mol. Cell Biol. Lipids.

[67] Borel P., Grolier P., Armand M., Partier A., Lafont H., Lairon D., Azais-Braesco V. (1996). Carotenoids in biological emulsions: Solubility, surface-to-core distribution, and release from lipid droplets. J. Lipid Res..

[68] Serrano J., Goñi I., Saura-Calixto F. (2005). Determination of β-carotene and lutein available from green leafy vegetables by an in vitro digestion and colonic fermentation method. J. Agric. Food Chem..

[69] Robert B., Horton P., Pascal A.A., Ruban A.V. (2004). Insights into the molecular dynamics of plant light-harvesting proteins in vivo. Trends Plant Sci..

[70] Granado-Lorencio F., Olmedilla-Alonso B., Herrero-Barbudo C., Pérez-Sacristán B., Blanco-Navarro I., Blázquez-García S. (2007). Comparative in vitro bioaccessibility of carotenoids from relevant contributors to carotenoid intake. J. Agric. Food Chem..

[71] Faulks R.M., Southon S. (2005). Challenges to understanding and measuring carotenoid bioavailability. Biochim. Biophys. Acta Mol. Basis Dis..

[72] Mène-Saffrané L., DellaPenna D. (2010). Biosynthesis, regulation and functions of tocochromanols in plants. Plant Physiol. Biochem..

[73] Kanwischer M., Porfirova S., Bergmüller E., Dörmann P. (2005). Alterations in tocopherol cyclase activity in transgenic and mutant plants of arabidopsis affect tocopherol content, tocopherol composition, and oxidative stress. Plant Physiol..

[74] Courraud J., Berger J., Cristol J.-P., Avallone S. (2013). Stability and bioaccessibility of different forms of carotenoids and vitamin A during in vitro digestion. Food Chem..

[75] Kopec R.E., Gleize B., Borel P., Desmarchelier C., Caris-Veyrat C. (2017). Are lutein, lycopene, and β-carotene lost through the digestive process?. Food Funct..

[76] Scheer L. (2009). Analytical sample preparation: The use of syringe filters. Filtr. Sep..

[77] Ortak M., Caltinoglu C., Sensoy I., Karakaya S., Mert B. (2017). Changes in functional properties and in vitro bioaccessibilities of β-carotene and lutein after extrusion processing. J. Food Sci. Technol..

[78] Rodriguez-Amaya D.B., Kimura M. (2004). HarvestPlus Handbook for Carotenoid Analysis.

[79] Eriksen J.N., Luu A.Y., Dragsted L.O., Arrigoni E. (2017). Adaption of an in vitro digestion method to screen carotenoid liberation and in vitro accessibility from differently processed spinach preparations. Food Chem..

[80] Corte-Real J. (2018). Bioavailability of Carotenoids—Impact of High Mineral Concentrations (BIOCAR).

[81] Galanakis C.M. (2017). Nutraceutical and Functional Food Components: Effects of Innovative Processing Techniques.

[82] Van der Ven C., Matser A.M., van den Berg R.W. (2005). Inactivation of soybean trypsin inhibitors and lipoxygenase by high-pressure processing. J. Agric. Food Chem..

[83] Mashurabad P.C., Palika R., Jyrwa Y.W., Bhaskarachary K., Pullakhandam R. (2017). Dietary fat composition, food matrix and relative polarity modulate the micellarization and intestinal uptake of carotenoids from vegetables and fruits. J. Food Sci. Technol..

[84] Halliwell B. (1990). How to characterize a biological antioxidant. Free Radic. Res. Commun..

[85] Mlček J., Juríková T., Škrovánková S., Paličková M., Orsavová J., Mišurcová L., Hlaváčová I., Sochor J., Sumczynski D. (2016). Polyphenol content and antioxidant capacity of fruit and vegetable beverages processed by different technology methods. Potravinarstvo.

[86] Chen X., Qin W., Ma L., Xu F., Jin P., Zheng Y. (2015). Effect of high pressure processing and thermal treatment on physicochemical parameters, antioxidant activity and volatile compounds of green asparagus juice. LWT Food Sci. Technol..

[87] Roldán-Marín E., Sánchez-Moreno C., Lloría R., de Ancos B., Cano M.P. (2009). Onion high-pressure processing: Flavonol content and antioxidant activity. LWT Food Sci. Technol..

[88] Butz P., Edenharder R., García A.F., Fister H., Merkel C., Tauscher B. (2002). Changes in functional properties of vegetables induced by high pressure treatment. Food Res. Int..

[89] Hsu K.-C. (2008). Evaluation of processing qualities of tomato juice induced by thermal and pressure processing. LWT Food Sci. Technol..

[90] Butz P., Fernández García A., Lindauer R., Dieterich S., Bognár A., Tauscher B. (2003). Influence of ultra high pressure processing on fruit and vegetable products. J. Food Eng..

